# The ecology of viruses in urban rodents with a focus on SARS-CoV-2

**DOI:** 10.1080/22221751.2023.2217940

**Published:** 2023-06-12

**Authors:** Adam M. Fisher, George Airey, Yuchen Liu, Matthew Gemmell, Jordan Thomas, Eleanor G. Bentley, Mark A. Whitehead, William A. Paxton, Georgios Pollakis, Steve Paterson, Mark Viney

**Affiliations:** a Department of Evolution, Ecology and Behaviour, Institute of Infection, Veterinary and Ecological Sciences, University of Liverpool, Liverpool, UK; b Department of Clinical Infection, Microbiology and Immunology, Institute of Infection, Veterinary and Ecological Sciences, University of Liverpool, Liverpool, UK; c Department of Infection Biology and Microbiomes, Institute of Infection, Veterinary and Ecological Sciences, University of Liverpool, Liverpool, UK; d Centre for Genomic Research, University of Liverpool, Liverpool, UK

**Keywords:** Urban, rodents, virus, virome, COVID, zoonosis, SARS-CoV-2

## Abstract

Wild animals are naturally infected with a range of viruses, some of which may be zoonotic. During the human COVID pandemic there was also the possibility of rodents acquiring SARS-CoV-2 from people, so-called reverse zoonoses. To investigate this, we sampled rats (*Rattus norvegicus*) and mice (*Apodemus sylvaticus*) from urban environments in 2020 during the human COVID-19 pandemic. We metagenomically sequenced lung and gut tissue and faeces for viruses, PCR screened for SARS-CoV-2, and serologically surveyed for anti-SARS-CoV-2 Spike antibodies. We describe the range of viruses that we found in these two rodent species. We found no molecular evidence of SARS-CoV-2 infection, though in rats we found lung antibody responses and evidence of neutralization ability that are consistent with rats being exposed to SARS-CoV-2 and/or exposed to other viruses that result in cross-reactive antibodies.

## Introduction

Most human infections are zoonotic, with the SARS-CoV-2/COVID-19 pandemic the most recent example. With the continued increase in the size of the human population and urbanization there is a continuing risk of further zoonoses [1], though while urban-adapted mammals are more parasite rich than mammal species as a whole, they are not a significantly greater source of zoonoses [2].

Infections of people can also infect animals, so-called reverse zoonoses. This is more likely to occur with substantial direct or indirect contact between people and animals and, with the context of human urbanization, possibly in urbanized commensal rodent pest species [3,4]. SARS-CoV-2 is primarily a respiratory disease of people but also infects gut tissue and viral RNA can be detected in faeces [5,6] and in wastewater [7,8], which are potential routes of transmission from people to rodents.

A wide range of RNA viruses are already known from rats (*Rattus norvegicus*) and mice (*Apodemus sylvaticus*) (Table 1) and a number of rodent species are predicted to be potential hosts of SARS-CoV-2 but to also harbour other coronaviruses [9–11] giving the potential for viral recombination including recombination of SARS-CoV-2 with other coronaviruses [12,13].

**Table 1.** A summary of the diversity and distribution of known zoonotic RNA viral families among *Rattus norvegicus* and *Apodemus sylvaticus*. Data were obtained from published articles included in the Database of Rodent-associated Viruses (DRodVir) [51].

Viral family	*Rattus norvegicus*		*Apodemus sylvaticus*	
	Number of viral species	Number of countries	Number of viral species	Number of countries
Coronaviridae	10	2	14	1
Arenaviridae	12	2	39	2
Astroviridae	11	1	125	2
Hantaviridae	326	17	646	17
Paramyxoviridae	91	4	80	6
Picornaviridae	81	5	86	7

SARS-CoV-2’s host range early in the human pandemic excluded rats (*R. norvegicus*) and mice (*M. musculus*), though deer mice (*Peromyscus maniculatus*) and Syrian hamsters (*Mesocricetus auratus*) were susceptible [14,15]. The principal determinant of host range is the sequence of the host species’ angiotensin-converting enzyme 2 (ACE2) [14]. SARS-CoV-2 has evolved, resulting in virus genotypes with new characteristics, including an altered host range [16]. Specifically, some SARS-CoV-2 variants of concern (VOC) are better able to infect laboratory mice compared with the original virus genotypes [17]. SARS-CoV-2’s host range has also been evolved experimentally: passage of human-derived SARS-CoV-2 in laboratory mice resulted in rapid evolution of viruses that better infected mice, compared with the initial human-derived virus, while maintaining the ability to infect through human ACE2 [18–20]. One can envisage that there will be continuing selection pressure on SARS-CoV-2 to infect non-human animal species that it commonly comes into contact with, with potential effects on future human infection [21].

A risk assessment of the likelihood of SARS-CoV-2 infecting rodents and of onward exposure to people conducted by the UK Department for Environment, Food and Rural Affairs concluded (i) with satisfactory confidence, that there was a high likelihood that a SARS-CoV-2 VOC could infect a commensal rodent, and (ii) with unsatisfactory confidence, that such rodent infections were unlikely to infect the general population but that there could be occupational exposure [22]. This risk assessment highlighted important knowledge gaps, including the (i) endogenous coronaviruses of rodents, (ii) risk of recombination of SARS-CoV-2 with other coronaviruses, (iii) selection pressure on SARS-CoV-2 to infect and transmit among rodents, and (iv) viral dose required to infect a rodent and the degree of viral shedding from any such infected animal. Recent work has sought evidence of SARS-CoV-2 in rats from sewers in Belgium, but the virus was not detected by PCR analysis, though there were antibodies that cross-reacted with SARS-CoV-2, but these did not neutralize virus *in vitro* [23]. Similarly, work with *R. norvegicus* and *R. tanezumi* in Hong Kong did not PCR-detect SARS-CoV-2, but one rat had anti-SARS-CoV-2 antibodies with some evidence that these could neutralize SARS-CoV-2 [24]. Studies of rats in New York, USA found molecular evidence of SARS-CoV-2 infection and of anti-SARS-CoV-2 IgG antibodies, though these were not neutralizing [25]. The work presented here further contributes to investigating commensal rodent species’ exposure and/or infection with SARS-CoV-2, where we were mindful of a number of possibilities of human-derived SARS-CoV-2 interacting with commensal rodents, ranging from full, long-lived infections, through shorter-term infections, to exposure that did not result in a patent infection.

## Material and methods

### Study species

We sampled rats (*R. norvegicus*) and mice (*A. sylvaticus*) during the COVID-19 pandemic from 15 June 2020 to 20 November 2020 in Liverpool, UK when B.1.389, B.1.177, and B.1.1.301 were the dominant SARS-CoV-2 strains present in England [26]. We trapped rats from an urban park and sewage treatment works; we trapped *A. sylvaticus* from urban parks (Supplementary Methods). These animals were used for viral metagenomic sequence analysis, PCR diagnosis of SARS-CoV-2, and serological analysis. An additional 41 rats (21 male, 17 female, 3 not determined) were obtained from southern England and used for serology (Supplementary Methods).

### RNA extraction

From each animal, we collected lung (all lobes) and small intestine tissue, and rectal faecal material from which we extracted RNA and pooled samples in three ways (Supplementary Methods).

### Positive controls

For the metagenomics and PCR we generated positive controls by spiking samples with high (10^8^) and low (10^4^) doses of SARS-CoV-2 virus (Supplementary Methods).

### Metagenomic sequencing and bioinformatics

We metagenomically sequenced the RNA following rRNA depletion and processed the data to classify reads using Kraken2 [27], using the standard and viral Kraken2 database, as described in the Supplementary Methods. We sought to assemble viral genomes, taking Kraken2-defined reads and attempted SPAdes genome assembly with default parameters [28] (Supplementary Methods). For reads Kraken2 putatively identified as SARS-CoV-2-derived, we Bowtie2-mapped them to the genome and compared with ARTIC primer locations (github.com/artic-network/primer-schemes), which was used in our laboratory and is a potential source of contamination.

### PCR for SARS-CoV-2

SARS-CoV-2 was amplified using the ARTIC V3 multiplex primer panel (artic.network; [29]). cDNA was synthesized with LunaScript RT SuperMix Kit and PCR-amplified with the Q5 High-Fidelity PCR Kit (both New England Biolabs).

## Quantitative analysis

We determined viral presence by expressing the number of Kraken2-identified reads for each virus as a proportion of all sequence reads in that sample. For all viruses, our detection cut-off was the proportion of SARS-CoV-2 reads returned by our 10^4^ positive control. We did this separately for rats and mice; all results presented here are after applying this threshold.

From this we measured the virus infections, separately for rats and mice as: (i) the number of viruses in each sample, and compared these between rats and mice using a generalized linear model with Poisson error correction, with host species a fixed effect and virus number the response variable; (ii) the prevalence of different viruses among the samples; (iii) the number of infections caused by different viruses, where we assigned each virus to a viral family and counted the number of animals so infected, expressed as a proportion of the total number of viral infections; (iv) the viral load of viruses, as the maximum number of sequence reads for that virus for any single host, expressed as a proportion of all the reads that we obtained for that host; and (v) for rats, the tissue association (lung, gut, faeces) of different viruses, focussing on the pools from 10 rats.

### Serology

We used ELISAs to assay for antibodies to the SARS-CoV-2 Spike protein, assaying tissue fluid extracts of heart, liver and lung tissue (Supplementary Methods). Our positive controls were laboratory rats or mice immunized with SARS-CoV-2 Spike protein, after [30] (Supplementary Methods). We validated using tissue fluid samples in three ways (Supplementary Methods).

We used heart tissue fluid and lung tissue fluid samples for IgG and IgA ELISAs, respectively, and report ELISA results as Optical Densities (OD) or as titres (Supplementary Methods).

### Neutralization assays

We tested the extent to which wild rat heart and rat lung tissue fluid could inhibit the ability of SARS-CoV-2 to infect mammalian cells, which we did using an *in vitro* neutralization assay, after [31] (Supplementary Methods).

## Results

### Animals

**Figure 1.**
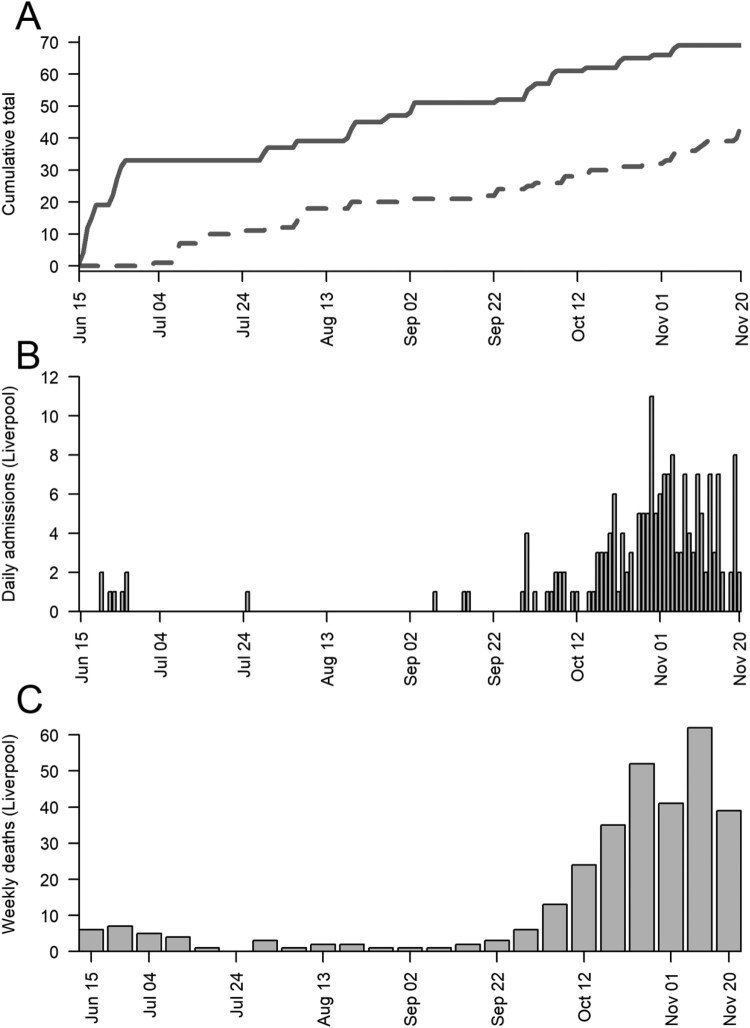
Between the 15 June 2020 and 20 November 2020 (A) the cumulative number of rats (dotted) and mice (solid) caught in Liverpool, (b) the daily number of COVID-19 hospital admissions in Liverpool (data from the National Health Service, UK) and (C) the weekly number of COVID-19 deaths in Liverpool (data from the Office for National Statistics, UK).

We trapped 45 rats, 10 (7 male, 3 female) from an urban park and 35 (14 male, 16 female, 5 unknown) from the sewage treatment works, and 69 mice (36 male, 33 female) from urban parks all in Liverpool. Trapping coincided with high and low prevalence of SARS-CoV-2 in people (Figure 1), giving temporal variation in the rodent SARS-CoV-2 exposure risk. A further 41 rats were obtained from southern England and used for serology (Supplementary Methods).

### Metagenomics

The 10^4^ viral dose difference between the high and low (10^8^ and10^4^, respectively) dose controls resulted in a 3.7 and 7.2 × 10^3^ (rat and mouse, respectively) magnitude difference in the proportionate number of reads recovered, so supporting using the proportionate number of sequence reads as a semi-quantitative, comparative measure of viral load.

After removing host reads, 1.7 and 3.5% of reads were Kraken2-classified as viral, for rats and mice, respectively. We detected 297 different viruses; 264 were only found in rats, 13 only in mice, and 20 in both (Supplementary Table 1). Rats and mice had an average 37.9 (95% CI, 31.4–45.3) and 4.3 (95% CI, 3.7–4.9) different viruses, respectively, which differed significantly (*df *= 68, *z *= 25.31, *p* < 0.001). In rats, 97 viruses were only found in lung, 7 only in gut tissue, and 4 only in faeces (Supplementary Table 2).

Rats and mice had different representation of viral families (Table 2). Four viral families (Picornaviridae, Baculoviridae, Poxviridae, Astroviridae) were in the top ten families shared between mice and rats. Coronaviridae accounted for 0.7% of rat viral infections; none occurred in mice.

**Table 2.** The percentage of all infections caused by viral species belonging to different viral families, for the 10 highest ranked named viral families, and Coronaviridae, showing the viral type and known host groups.

Rat			Mouse		
Family	Notes	Percent	Family	Notes	Percent
Picornaviridae	RNA; vertebrates	16	Peribunyaviridae	RNA; animals	42
Unclassified	–	12	Baculoviridae	DNA; arthropods	20
Virgaviridae	RNA; plants	9	Poxviridae	DNA; animals	8
Herpesviridae	DNA; vertebrates	7	Astroviridae	DNA; vertebrates	7
Baculoviridae	DNA; arthropods	6	Picornaviridae	RNA; vertebrates	5
Poxviridae	DNA; animals	6	Picobirnaviridae	RNA; mammals	5
Nodaviridae	RNA; animals	6	Hantaviridae	RNA; mammals	4
Retroviridae	RNA; animals	4	Unclassified	–	3
Dicistroviridae	RNA; invertebrates	4	Phenuiviridae	RNA; mammals	2
Astroviridae	DNA; vertebrates	4	Adenoviridae	DNA; vertebrates	1
Flaviviridae	RNA; vertebrates	4	Anelloviridae	DNA; vertebrates	1
Coronaviridae	RNA; vertebrates	0.7	Coronaviridae	RNA; vertebrates	0

In mice, three viruses (Shamonda orthobunyavirus, Simbu orthobunyavirus, Choristoneura fumiferana granulovirus) had a greater than 50% prevalence, whereas in rats there were 23 viruses with a greater than 50% prevalence, with 4 each belonging to the Virgaviridae and Picornaviridae, and 3 to the Nodaviridae family (Supplementary Table 3).

Considering viral load, in rats 4 viruses each accounted for more than 0.5% of reads; in mice three viruses each accounted for more than 1% of reads (Supplementary Table 4). Viruses with this large number of reads were also among the most prevalent viruses.

### Coronaviridae and SARS-CoV-2

Three rat samples had SARS-CoV-2 sequence reads above the defined cut-off; specifically, 1 lung tissue pool from 3 rats, 2 gut tissue pools each from three different rats (together encompassing 7 urban park rats). Aligning these reads to the SARS-CoV-2 genome showed that 8, 39, and 72 read pairs aligned (compared to 72 for the low dose positive control), and their starting positions typically corresponded to ARTIC SARS-CoV-2 scheme primer sites, which was used in our laboratory in the UK COVID surveillance programme. Thus, these reads likely represent low level contamination. Reads from the positive controls had a uniform pattern of alignment across the genome. No reads aligned to the SARS-CoV-1 genome. Together, these data indicate that we did not detect SARS-CoV-2 infection in rats. No mouse samples had SARS-CoV-2 reads above the defined cut-off.

### Other Coronaviridae

We detected other Coronaviridae in rats; specifically Ferret coronavirus in 7 samples (4 individuals and 3 lung tissue pools each from 3 or 4 rats); Rhinolophus bat coronavirus HKU2 in one lung tissue pool from 3 rats, and Middle East respiratory syndrome-related coronavirus in 7 rat samples (6 individuals and 1 gut tissue pool from 3 rats). We were unable to assemble genome sequence of these three viruses.

We did not detect Coronaviridae in mice, but many mouse samples had substantial numbers of reads from an Avian coronavirus. No single mouse sample had reads above our cut-off, though by halving this, then 9 mouse samples were positive (5 individuals and 2 pools of pairs of mice).

### Hantaviridae

We found evidence of hantavirus infection; specifically, Oxbow orthohantavirus in 16 rat samples (10 individuals, 3 lung tissue pools, and 3 gut tissue pools each from 3 or 4 rats) and in 6 individual mouse samples; and Seoul orthohantavirus in one rat. We partially assembled the Seoul orthohantavirus sequence, with the largest contig being 4.8 kb (N50 1.5 kb) and BLAST analysis confirmed Seoul orthohantavirus identity.

### SARS-CoV-2 PCR

We did not PCR-detect SARS-CoV-2 in any rats or mice. Our high positive control strongly amplified, our low positive control amplified less strongly, and our negative controls did not amplify. This is further evidence of the absence of active SARS-CoV-2 in rats and mice.

### Serology

We detected immunoglobulin in tissue fluid samples. Specifically, rat negative control heart and liver tissue fluid contained 166 and 437 μg/mL total IgG, respectively, compared with 6,770 μg/mL in serum, thus heart and liver samples are 2.4 and 6.4% of the serum IgG concentration. A randomly selected wild rat had 670 and 400 μg/mL concentration in heart and liver, respectively, the same order of magnitude as laboratory animals. For anti-SARS-CoV-2 Spike protein antibodies, in positive control rats the titre was 640,000 in serum, and 32,000 in liver and heart tissue fluid, which are compatible with results for the total IgG concentration. Together these results show that IgG and antigen-specific antibodies can be detected in tissue fluid, though the concentration is lower compared with serum. For total IgA in positive control rats, the serum concentration was 301 μg/mL, but 28, 7.9 and 1.5 μg/mL in lung, heart and liver tissue fluid, respectively. In two wild rats, lung tissue had high total IgA concentrations (307 and 1188 μg/mL), likely due to the high, wild antigenic exposure [32]. These results show that it is possible to detect IgA in lung tissue fluid. Based on these results we screened rat and mouse heart tissue fluid for anti-SARS-CoV-2 Spike IgG antibodies, and rat lung tissue fluid for anti-SARS-CoV-2 Spike IgA antibodies.

**Figure 2.**
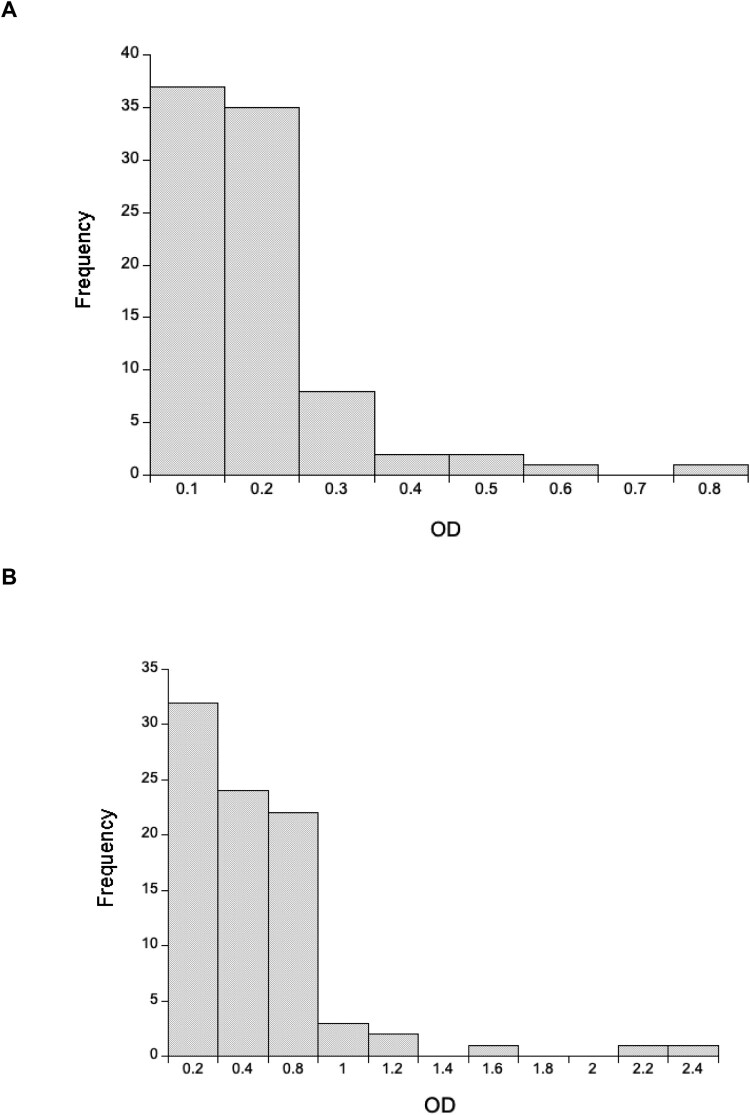
The distribution of optical densities (OD) of anti-SARS-CoV-2 (A) IgG and (B) IgA responses for 86 wild rats for 1:640 and 1:20 diluted heart and lung tissue fluid, respectively. The OD of the positive control tissue fluid is 3.55 for IgG and 0.825 for IgA.

Using a 1:640 dilution of rat heart tissue, we detected very low concentrations of anti-SARS-CoV-2 Spike IgG antibodies, with wild rat ODs less than a third of the positive control (Figure 2). Using a 1:20 dilution of lung tissue, we similarly found that most animals had very low concentrations of anti-SARS-CoV-2 Spike IgA antibodies, though 7 rats (6 southern England, 1 Liverpool) had ODs greater than or equal to the positive control (Figure 2). In interpreting this it is important to remember that the positive controls were immunized intramuscularly, not in the lung, and so this control does not maximize the lung IgA response. Notwithstanding, the high OD of 7 rats is consistent with exposure to SARS-CoV-2 and/or other coronaviruses that result in antibodies that recognize SARS-CoV-2 Spike protein. For these 7 rats with high IgA ODs their mean (±SD) IgG OD was 0.130 ± 0.0029, which compares with a mean of 0.141 ± 0.116 of all 86 rats, which suggests that these putatively IgA positive rats do not have similarly high concentrations of IgG anti-SARS-CoV-2 antibodies.

**Figure 3.**
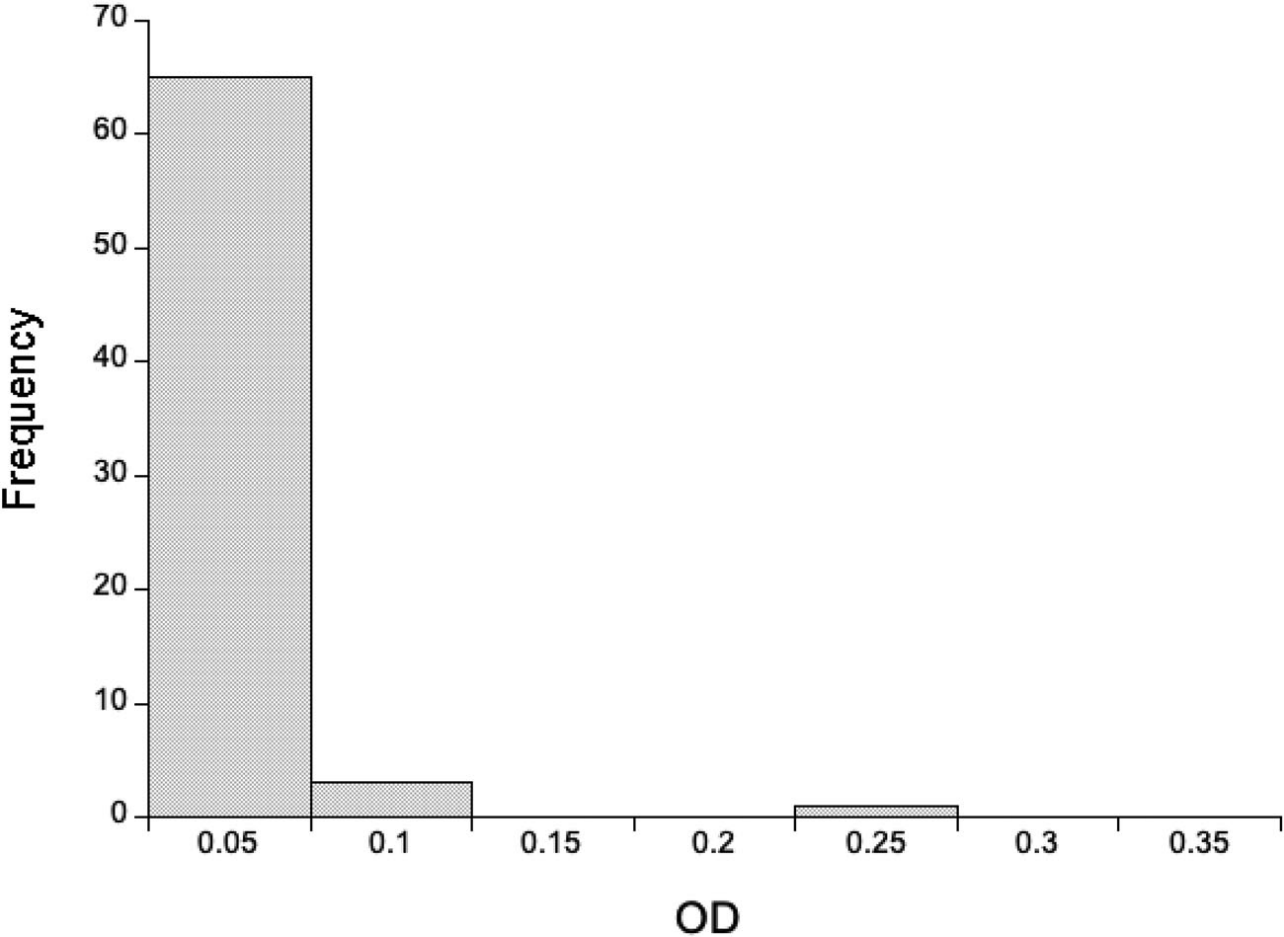
The distribution of optical densities (OD) of anti-SARS-CoV-2 IgG responses for 69 wild mice for 1:160 diluted heart tissue fluid. The OD of the positive control heart tissue fluid is 0.532.

Using a 1:160 dilution of mouse heart tissue, we detected very low concentrations of anti-SARS-CoV-2 Spike IgG antibodies, with the OD of the wild rats generally a tenth of the positive control (Figure 3). In interpreting this it is important to remember that the positive control is from *M. musculus*, while the wild mice are *A. sylvaticus*, but that the ELISA used *M. musculus* reagents.

### Neutralization assays

**Figure 4.**
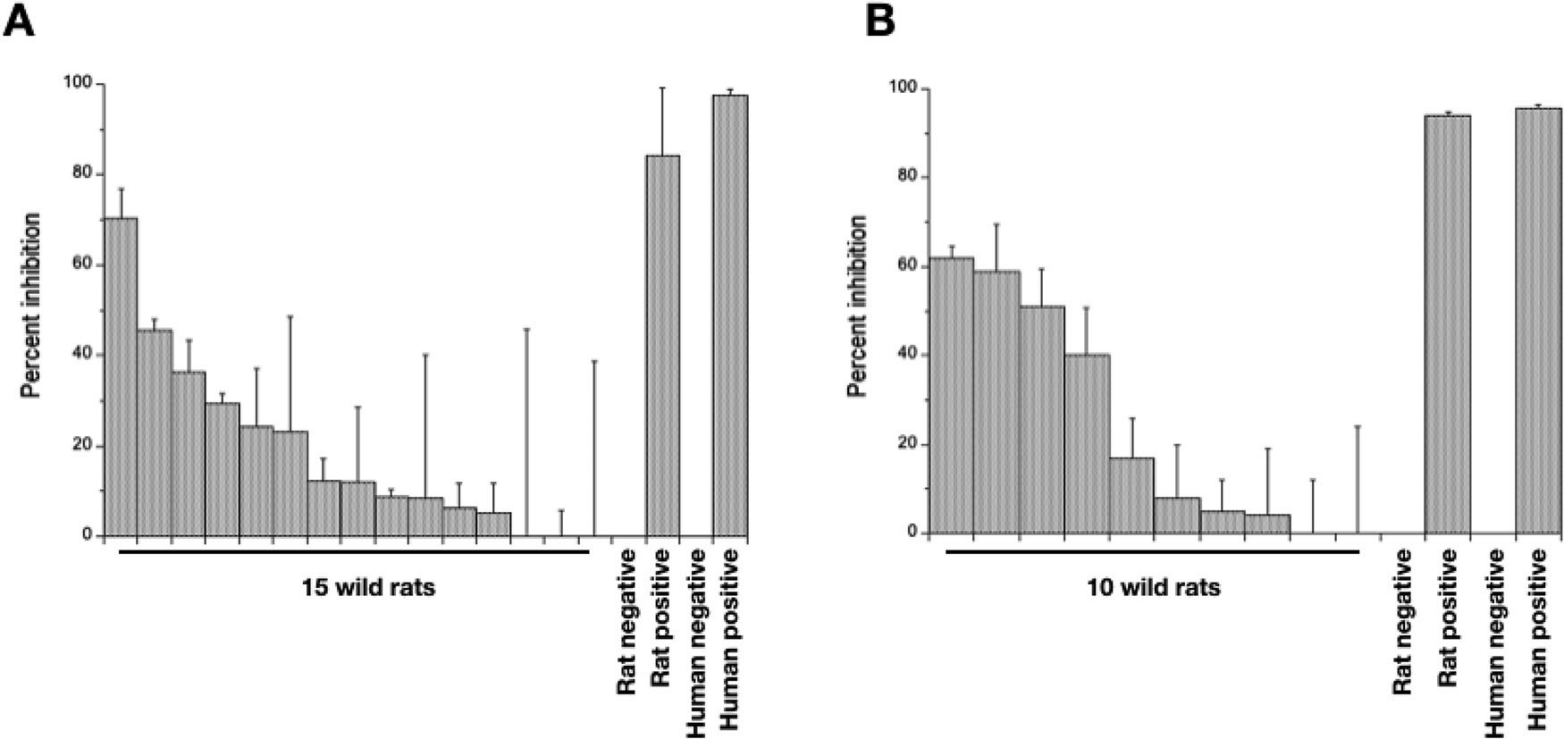
The mean percent inhibition of wild rat (A) heart and (B) lung tissue fluid at a 1 in 16 and 1 in 8 dilution, respectively. Errors bars are +1 SD. Rat and human positive and negative controls are as described in the Materials and Methods.

Three (of 15) rat heart tissue samples (at the highest concentration) partially neutralized pseudovirus particle infection (36–70% inhibition), compared with 84% positive control neutralization (Figure 4). Four rat lung samples (at the highest concentration) achieved 39–61% neutralization, compared with 93% positive control neutralization (Figure 4); three of these samples were putatively IgA positive samples. Of note, lung and heart tissue from a single rat achieved 59 and 23% neutralization, respectively. These results are consistent with wild rats being exposed to SARS-CoV-2 and/or other coronaviruses that generates an immune response that has the ability to neutralize infection *in vitro*.

## Discussion

We have investigated the viruses of urban rats and mice and their exposure and/or infection with SARS-CoV-2 as a reverse zoonosis during the 2020 human SARS-CoV-2 pandemic. We were mindful of a range of scenarios, from full, long-lived infections in rodents, through short-term infections, to exposure not resulting in a patent infection. Metagenomics and PCR of lung, gut, and faeces and serological analysis finds some evidence consistent with rats being exposed to SARS-CoV-2. We found no such evidence for mice.

For rats, the evidence is (i) lung anti-SARS-CoV-2 Spike IgA responses equal to or greater than our positive control and (ii) that lung and heart tissue fluid can partially neutralize SARS-CoV-2 PVP *in vitro*. Our interpretation of these data is that rats are being exposed to SARS-CoV-2, perhaps resulting in short-lived infections that result in detectable anti-SARS-CoV-2 antibodies, where lung exposure results in lung IgA responses, but not systemic IgG responses. The alternative interpretation is that the IgA antibodies were generated in response to other viral infections and that these antibodies cross-react with SARS-CoV-2 Spike protein and can partially neutralize it. Recent analysis of publicly-available SARS-CoV-2 sequence data conclude that there is human to animal (cat, dog, mink, deer) transmission, and that this is likely more common than animal to human transmission [33].

Our results are consistent with studies of rats in Belgium, Hong Kong and New York that found evidence of antibodies recognizing SARS-CoV-2 and with some evidence of their neutralization ability [23–25]. Our approach goes further by considering lung IgA responses, which may be more appropriate in understanding rodent exposure to SARS-CoV-2 compared with serum IgG.

Serological analyzes are potentially very useful for detecting short-lived, low dose SARS-CoV-2 infection, because any immune responses will persist life-long. Two challenges with interpreting serological data are (i) cross-reaction of antibodies produced against other non-SARS-CoV-2 viruses, producing false positives, (ii) the absence of appropriate serological positive controls. Our positive controls were laboratory animals immunized with purified SARS-CoV-2 Spike protein in adjuvant, twice, intramuscularly, resulting in a high anti-SARS-CoV-2 antibody titre, but one unlikely ever be achieved naturally. A better positive control, appropriate to the type of infection we hypothesize may occur in wild rodents, would be a pulmonary or enteric infection of low viral dose.

A UK DEFRA risk assessment of SARS-CoV-2 infection of rodents concluded with satisfactory evidence that there was a high likelihood that SARS-CoV-2 VOC could infect a commensal rodent, and the data we provide here is potential evidence of this.

We also used metagenomics to identify other viruses in rats and mice, where we used our low dose positive control to provide an objective cut-off for determining a positive signal of viral infection. We found evidence of almost 300 viruses, principally in rats, and then mainly from lung tissue. Our Kraken2-based analysis is good initial evidence of viral identity, though assembly of viral genome sequence is better evidence, so our viral identification should be considered indicative rather than definitive. We found viruses from a wide range of viral families, with the most highly represented families differing between rats and mice. These results are broadly comparable to other molecular surveys of wild rodent viruses, including: in the US a range of viruses already known from mammals (including members of the Coronaviridae, Astroviridae, Picornaviridae, Picobirnaviridae, Adenoviridae, Papillomaviridae, Parvovirinae and Circoviridae) as well as viruses known from insects and plants; in gut samples from rats in Berlin, Germany a wide range of viruses [34,35].

We found evidence of other coronavirus infections; specifically, in rats of a betacoronavirus, Middle East respiratory syndrome-related coronavirus (MERS) and two alphacoronaviruses; in mice a gammcoronavirus. Coronaviruses have previously been detected in wild rodents, including: alphacoronaviruses in UK rats (but not in *Mus* spp. or *Apodemus* spp.) [36,37]; betacoronaviruses in *Apodemus* sp. in France [11]; alphacoronaviruses in rodents in the Congo basin [38]; alpha and betacoronaviruses in *Apodemus* spp. and *Rattus* spp. in China [39,40]; coronaviruses in house mice (*M. musculus*) but not in *Rattus* spp. in the Canary Islands [41], and a high seroprevalence against coronaviruses in US *R. norvegicus* [42]. Together, this shows that a range of coronaviruses, including betacoronaviruses, do occur in wild rodents, which contributes to addressing the DEFRA-identified knowledge gaps of the endogenous coronaviruses of rodents. The potential for recombination of SARS-CoV-2 with such endogenous coronaviruses (also a DEFRA-identified knowledge gap) remains unknown, but the data we present is supportive of rats being potentially exposed to SARS-CoV-2 that are infected with other coronaviruses, which is the necessary prelude to any potential recombination.

Concerning our putative identification of MERS in rats, MERS is related to a number of bat coronaviruses and to a hedgehog coronavirus [43]. The hedgehog coronavirus is relatively common in hedgehogs in Europe with a prevalence ranging from 10 to 58% [44]. The Kraken2 database we used to identify our metagenomic sequence reads contains both MERS, hedgehog coronavirus, and other bat coronaviruses. On balance, we suspect that our rat MERS viral reads are more likely to be derived from a hedgehog coronavirus, or perhaps a bat coronavirus, rather than *sensu stricto* MERS since (i) both hedgehogs and bats live within the environments where we caught rats and (ii) that MERS has only been reported to infect humans, bats and camels [45]. The other alternative is that these rats MERS-assigned reads are derived from a hitherto unknown betacoronavirus. To resolve this, definitive identification of the rat virus would be needed, which would require viral genome assembly, which we were unable to achieve. Notwithstanding, the discovery of an additional betacoronavirus in rats strengthens the idea of recombination among rat betacoronavirsues, which address DEFRA-identified knowledge gaps.

We found evidence of hantavirus infection in rats and mice, specifically of Oxbow othohantavirus in rats and mice and Seoul orthohantavirus in a rat. Hantaviruses are common in rodent hosts and can result in human infection and disease [46]. Seoul virus has a worldwide distribution and in the UK has been found in wild and pet rats [47–50]; our report is consistent with these. Oxbow orthohantavirus was first described from an American shrew mole (*Neurotrichus gibbsii*), after which there are no further reports. We have evidence that this virus is widespread in rats and mice (*A. sylvaticus*), and if substantiated this would be a significant extension of its known host and geographical range.

In conclusion, we present evidence consistent with rats being exposed to SARS-CoV-2, possibly from a human source, and/or other coronaviruses resulting in antibodies that cross-react with SARS-CoV-2 and which partially neutralizes it. If substantiated, this could have important implications for the future evolutionary trajectory and epidemiology of SARS-CoV-2 and for the future risk of viral recombination with potential risk for animal and human populations.

## Data deposition

The metagenomic sequence data are deposited in the European Nucleotide Archive as accessions PRJEB53828 and PRJEB5329.

## Supplementary Material

Supplemental Material
