# Supplementary material for: The ecology of viruses in urban rodents with a focus on SARS-CoV-2
Source: Emerg Microbes Infect. 2023 Jun 12;12(1):2217940. doi: 10.1080/22221751.2023.2217940 (PMC10262798; doi:10.1080/22221751.2023.2217940)
Supplement: Supplemental Material [file TEMI_A_2217940_SM7004.zip › Supplementary Methods.docx]

**Supplementary Methods**

**The Ecology of Viruses in Urban Rodents with a Focus on SARS-CoV-2**

**Material and Methods**

***Study species***

We live trapped rats from an urban park (Greenbank Park) and a sewage treatment works (United Utilities, Liverpool Docks); we trapped *A. sylvaticus* from urban parks (Sefton Park, Princes Park, Calderstones Park and Wavertree Botanic Gardens). An additional 41 rats (21 male, 17 female, 3 not determined) from southern England pest controllers (in Reading, 51.466479, -0.966974, and Basingstoke, 51.278179, -1.065996) were used for serology.

***RNA extraction***

RNA was then separately extracted from 20 – 30 mg of these samples using the Qiagen RNeasy Mini Kit Plus following the manufacturer’s protocol and RNA samples were stored at -80^o^C.

***Pooling RNA samples***

For metagenomic sequencing and PCR analysis we pooled RNA samples in the following way: (1) For the 10 rats caught in urban parks we pooled RNA from the same tissue type (lung, gut, or faeces) from three or four rats, with equal amounts of RNA from each rat. Pooling in this way would tell us about the tissue sample-associations of any viruses; (2) For the 35 rats caught in the sewage treatment works we pooled the three different samples (lung, gut, faeces) of each animal, with equal amounts of RNA from each sample. Pooling in this way would tell us about the prevalence of any viruses in the rat population. (3) For the 69 mice, the RNA was pooled as 2 (above) and for 35 mice this pooled RNA was sequenced for individual mice; for 34 mice RNA from randomly selected pairs of mice caught on the same day was pooled.

***Positive controls – metagenomic sequencing and PCR***

For the PCR and metagenomic sequence analysis we generated positive controls by spiking tissue samples with inactivated SARS-CoV-2. Specifically, we took samples of tissue from gut, lung and faeces (as above) from laboratory SPF rats and mice (*Mus musculus*). To each of these individual samples we then generated two controls: a ‘high’ control of 10^8^ and a ‘low’ control of 10^4^ UV inactivated SARS-CoV-2 virus particles (grown in Vero E62 cells) per gram of tissue. The 10^4^ dose is one that was used to successfully infect *Peromyscus maniculatus* with SARS-CoV-2 (Fagre *et al.,* 2021). These samples were then processed for RNA extraction (as above) and the RNA samples then pooled as for the rats caught in the sewage treatment works, separately for the SPF rats and mice.

***Metagenomic sequencing and bioinformatics***

The RNA was rRNA depleted using the NEBNext rRNA process using human / mouse / rat baits used with the rat-derived samples, and human / mouse / rat and bacterial baits with the mouse-derived samples, following the manufacturer’s protocol. Depleted material was then purified using Ampure RNA XP beads and successful rRNA depletion was confirmed using a fragment length analyser. Depleted RNA was then used in the NEBNext Ultra II Directional RNA Library Kit for Illumina, with a fragmentation time of 7 minutes, and after 12 cycles of amplification the libraries were purified using Ampure XP beads. Each library was quantified using Qubit and the size distribution assessed. Final libraries were pooled in equimolar amounts and the quantity and quality of the pool was assessed with a Bioanalyzer and subsequently by qPCR using the Illumina Library Quantification Kit from Kapa on a Roche Light Cycler LC480II. Template DNA was diluted to 300 pM and denatured for 8 minutes at room temperature using freshly diluted 0.2 N sodium hydroxide, and the reaction terminated by the addition of 400 mM TrisCl pH 8. The libraries were sequenced on the Illumina NovaSeq 6000 platform following a standard workflow over 8 lanes of an S4 flow cell generating 2 x 150 bp paired-end reads.

Initial processing and quality assessment of the sequence data was performed using an in-house pipeline. Briefly, base-calling and de-multiplexing of indexed reads was performed by CASAVA version 1.8.2 (Illumina) to produce sample sequence files in FASTQ format that were then trimmed to remove Illumina adapter sequences using Cutadapt version 1.2.1. The reads were further trimmed to remove low quality bases, using Sickle version 1.200 with a minimum window quality score of 20. After trimming, reads shorter than 20 bp were removed and if both reads from a pair passed this filter, each was included in the R1 (forward reads) or R2 (reverse reads) file; if only one of a read pair passed this filter, it was included in the R0 (unpaired reads) file.

We next removed host (*R. norvegicus*, *A. sylvaticus, M. musculus*) sequence reads from the adapter and quality-trimmed paired end read data where reference genomes were indexed and aligned with **Bowtie2** (Langmead & Salzberg, 2012) and unmapped reads (*i.e.* reads that did not align to the host reference) were extracted. We then classified remaining reads using Kraken2 (Wood *et al*., 2019), using both the standard and viral **Kraken2** database. A custom script was used to combine the **Kraken2** reports into taxonomy abundance tables at different taxonomic levels.

We sought to assemble some viral genomes, which we did by taking the relevant Kraken2-defined reads and attempted genome assembly using **SPAdes** with default parameters (Bankevich *et al*., 2012).

***Serology***

We assayed tissue fluid extracts of heart, liver and lung tissue for ELISA. To do this, 0.5 g of each tissue from the wild caught rats and mice was homogenised in 1 mL of phosphate buffered saline, supplemented with Triton to a final concentration of 1 % w/v and a protease inhibitor cocktail (Sigma) to a final concentration of 20% v/v, left on ice for 30 minutes, centrifuged at 13,000 *g* for 10 minutes at 4^o^C, and then the supernatant removed, which was then stored at -20^o^C.

# For positive control samples Wister rats and C57BL/6J mice were immunised intramuscularly with 5 μg SARS-CoV-2 Spike protein (residues 319-541) (Thermo Fisher) administered in TiterMax Gold Adjuvant on day 0 and on day 50. Animals were killed on day 58 and serum prepared and stored at -20^o^C, and tissue dissected and tissue fluid samples prepared as described above. We used non-immunised laboratory SPF rats and mice (*M. musculus*) as negative controls.

# We validated the use of tissue fluid samples for ELISA in three ways. Firstly we determined the concentration of total IgG in (i) negative control laboratory rats, comparing serum, liver and heart tissue fluid samples, and (ii) a sub-sample of wild rat liver and heart tissue fluid samples. The total IgG assays were conducted for rats and mice using the IgG Total Rat Uncoated ELISA Kit (Thermo Fisher), following the manufacturer’s protocol. Secondly, we determined the concentration of anti-SARS-CoV-2 Spike IgG in positive control Spike protein immunised rats, comparing serum, liver and heart tissue fluid samples, using the relevant ELISA protocol described below. Thirdly, we determined the concentration of total IgA in (i) positive control Spike protein immunised rats, comparing serum, lung, liver, and heart tissue fluid, and (ii) a sub-sample of wild rat lung tissue fluid samples.

For the IgG anti-SARS-CoV-2 Spike protein ELISA, plates were coated with SARS-CoV-2 Spike protein (Thermo Fisher) at a concentration of 1 μg / mL overnight at 4^o^C. All buffers used were supplied by Thermo Fisher and used as recommended. After coating, plates were washed twice in wash buffer, blocked with blocking buffer for 2 hours at room temperature, then washed twice with wash buffer. Samples were diluted in assay buffer and titrated in doubling dilutions on plates, then left for 2 hours with shaking at room temperature, after which they were washed four times in wash buffer, after which a 1 : 5,000 dilution (in assay buffer) of goat anti-rat IgG horseradish peroxidase conjugate (Thermo Fisher) was added, which was incubated for 1 hour with shaking at room temperature, then washed four times with wash buffer before the addition on 100 μL of TMB substrate solution, which was then stopped after 25 minutes with stop solution. The mouse ELISAs were done in the same fashion except that goat anti-mouse IgG horseradish peroxidase conjugate (Thermo Fisher) diluted 1 : 10,000 in assay buffer was used, and that once the substrate was added the assay was stopped after 20 minutes.

We used rat lung tissue fluid samples for IgA anti-SARS-CoV-2 Spike protein ELISAs. These were conducted as described for the IgG ELISAs except that the detection antibody was goat anti-rat IgA horseradish peroxidase conjugate (Invitrogen) diluted in assay buffer at 1 : 10,000 and the reaction developed for 15 minutes before being stopped.

Note that the target mouse species is *A. sylvaticus* but the murine kits and regents are designed for use with the mouse *M. musculus.* Previous work (*e.g.* Jackson *et al*., 2009; Clerc *et al.,* 2019) has shown cross-reactivity of antibodies between these two mouse species.

We report the ELISA results as Optical Densities (OD) or as titres. When presenting ODs we do so for a dilution of the sample where that dilution placed the ELISA data in the non-asymptotic part of the dilution *vs.* OD relationship. Titre is the reciprocal of the dilution of the serum or tissue fluid sample that achieves an OD greater than the negative in the assay, where we defined negative as the mean + 2 x standard deviations of the OD of our negative control sample.

***Neutralization assays***

SARS-CoV-2 enveloped pseudo-typed virus particles (PVP) displaying the ancestral, Wuhan Spike protein (Accession MN908947) were generated by transfecting HEK293T Lentix cells with a pCSFLW lentiviral luciferase reporter, the SARS-CoV-2 envelope expression plasmid, and the lentiviral backbone p8.91 (Carnell *et al*., 2017; Di Genova *et al*., 2021). Samples were serially diluted, added to PVP and held at room temperature for 30 minutes. Next, this virus / sample dilution mix was used to infect HEK293T ACE2 TMPRSS2 cells and PVP infection was monitored by luciferase activity, where (i) 0 % inhibition was taken as the infection values of the virus in the absence of human convalescent plasma included in each experiment, and (ii) 0 % inhibition as the infection values of two consecutive high dilutions not inhibiting virus entry. We used 15 rat heart samples that represented the range of ELISA IgG ODs that we observed; we used 10 rat lung samples that included 7 ELISA IgA putatively positive samples, and 3 putatively negative samples. The tissue fluid samples were prepared as described above for the serological analysis, except that they were prepared in phosphate buffered saline only and inactivated at 56 °C for 30 mins to destroy complement or residual virus. Samples were assayed in a doubling dilution series starting from a 1 in 8 and 1 in 16 dilution for the lung and heart samples, respectively. Rat positive controls were heart and lung tissue fluid prepared from positive control SARS-CoV-2 Spike protein immunised rats (above); rat negative controls were heart and lung tissue fluid prepared from unimmunised laboratory rats. The human positive controls was serum for an individual who had had multiple SARS-CoV-2 vaccinations; the human negative control was serum from the same individual before SARS-CoV-2 vaccination.

**References**

Bankevich, A., Nurk, S., Antipov, D., Gurevich, A.A., Dvorkin, M., Kulikov, *et al.* 2012. SPAdes: a new genome assembly algorithm and its applications to single-cell sequencing. *J. Comp. Biol*. 19, 455-477.

Carnell, G., Grehan, K., Ferrara, F., Molesti, E., Temperton, N. 2017. An optimized method for the production using PEI, titration and neutralizationof SARS-CoV Spike Luciferase Pseudotypes. Bio Protoc. 7, e2514.

Clerc ,M., Babayan, S.A., Fenton, A., Pedersen, A.B., 2019. Age affects antibody levels and anthelmintic treatment efficacy in a wild rodent. Int. J. Parasitol. Parasites Wildl. 8, 240-247.

Di Genova, C., Sampson, A., Scott, S., Cantoni, D., Mayora-Neto, M., Bentley, E., *et al.* 2021 Titration, neutralisation, storage and lyophilisation of Severe Acute Respiratory Syndrome Coronavirus 2 (SARS-CoV-2) Lentiviral Pseudotypes. Bio Protoc., 11, e4236.

Fagre, A., Lewis, J., Eckley, M., Zhan, S., Rocha, S.M., Sexton, N.R., *et al*., 2021. SARS-CoV-2 infection, neuropathogenesis and transmission among deer mice: Implications for spillback to New World rodents. PLoS Pathog. 17, e1009585.

Jackson, J.A., Friberg, I.M., Bolch, L., Lowe, A., Ralli, C., Harris, P.D., *et al.*, 2009. Immunomodulatory parasites and toll-like receptor-mediated tumour necrosis factor alpha responsiveness in wild mammals. BMC Biol. 7, 16.

Langmead B, Salzberg S. 2012. Fast gapped-read alignment with Bowtie 2. Nat. Meth. 9, 357-359.

Wood, D. E., Lu, J., Langmead, B., 2019. Improved metagenomic analysis with Kraken 2. Genome Biol. 20, 257.
